# Human Activity Recognition Based on Residual Network and BiLSTM

**DOI:** 10.3390/s22020635

**Published:** 2022-01-14

**Authors:** Yong Li, Luping Wang

**Affiliations:** 1 School of Biomedical Engineering, Sun Yat-sen University, Guangzhou 510006, China; liyong67@mail2.sysu.edu.cn; 2 School of Electronics and Communication Engineering, Sun Yat-sen University, Guangzhou 510006, China

**Keywords:** human activity recognition, residual network, BiLSTM, inertial measurement unit

## Abstract

Due to the wide application of human activity recognition (HAR) in sports and health, a large number of HAR models based on deep learning have been proposed. However, many existing models ignore the effective extraction of spatial and temporal features of human activity data. This paper proposes a deep learning model based on residual block and bi-directional LSTM (BiLSTM). The model first extracts spatial features of multidimensional signals of MEMS inertial sensors automatically using the residual block, and then obtains the forward and backward dependencies of feature sequence using BiLSTM. Finally, the obtained features are fed into the Softmax layer to complete the human activity recognition. The optimal parameters of the model are obtained by experiments. A homemade dataset containing six common human activities of sitting, standing, walking, running, going upstairs and going downstairs is developed. The proposed model is evaluated on our dataset and two public datasets, WISDM and PAMAP2. The experimental results show that the proposed model achieves the accuracy of 96.95%, 97.32% and 97.15% on our dataset, WISDM and PAMAP2, respectively. Compared with some existing models, the proposed model has better performance and fewer parameters.

## 1. Introduction

HAR has received a lot of attention in recent years for its applications in smart homes, fall detection for the elderly, sports training, medical rehabilitation, and misbehavior recognition [1,2]. For example, by analyzing the movements of elderly people living alone, the fall behavior can be detected for seeking help from family members in time. Fitness people can obtain their own exercise data by counting steps and recognizing exercise status to achieve scientific exercise and fitness management. Doctors can diagnose patients with knee diseases by gait analysis. In the rehabilitation phase, the rehabilitation plan can be adjusted based on the movement data of patients with lower limb diseases. The HAR technologies could be divided into two categories: camera-based and sensor-based [3,4]. The camera-based method extracts the human activity features from the video stream by placing a camera in the human surroundings. Although this approach can visually display the details of human action, it suffers from privacy issues, and its performance is subject to the quality of background illumination. On the contrary, the sensor-based approach has many advantages. It is unaffected by the surrounding environment and is promising to obtain higher accuracy. In addition, it will not cause privacy problems of users. Therefore, sensor-based approaches are more suitable for human activity recognition [5]. In this paper, we mainly discuss the problem of sensor-based HAR.

In the existing studies, researches placed the smart device on the waist [6], pants pocket [7], or wrist [8], using the inertial measurement unit (IMU) in the smart device to collect human activity data. The IMU including the accelerometer and gyroscope is used to measure human body acceleration and angular rate. Firstly, the sensor data are preprocessed for noise reduction and normalization. Then, the feature extraction and feature classification are performed to complete the human activity classification. In the past, magnificent progress has been made by conventional machine learning algorithms on HAR such as support vector machines (SVM), random forest (RF), and hidden Markov models (HMM). In [9], a k-nearest neighbor (KNN) model was used to classify human action, but some similar activities could not be distinguished. The paper [10] compared the performance of three classifiers, KNN, SVM, and RF, and the RF has the highest accuracy. Wang et al. [11] used extreme learning machine to recognize eight activities and achieved an accuracy of 70%. Duc et al. [12] designed a HAR system based on SVM by extracting 248 features to recognize six activities. Even though these methods achieve good results in some datasets, certain human experience is required for extracting hand-crafted features which results in a limited accuracy [6].

Unlike the traditional machine learning, deep learning (DL) can process the preprocessed IMU data without extracting hand-crafted features and is widely used in the activity recognition [13,14]. In recent years, many CNN-based HAR methods which extracted features automatically have been proposed [15,16,17]. In [18], a deep convolutional neural network is proposed to perform an effective HAR. Ignatov [15] proposed a CNN model for local feature extraction along with statistical features to obtain the global properties of the sensor time series. The recognition accuracy of his method on the public dataset WISDM is 93.32%. Huang et al. [17] proposed a two-stage end-to-end convolutional neural network to solve the problem of low accuracy of going upstairs and going downstairs. The model was tested on WISDM. Compared with the single-stage CNN, the recognition accuracy of going upstairs and going downstairs is improved. Alemayoh et al. [6] proposed a double-channel CNN to identify human behavior by accelerometer and gyroscope in the smartphone strapped to the waist. The accuracy reached 97.08%, but the network could overlook the temporal features. Qi et al. [19] proposed a fast and robust deep convolutional neural network to identify 12 complex human activities collected from a smartphone and the accuracy reached 94.18% in the experiment.

However, the activity recognition is a classification problem based on time series. CNN is hard to extract the long-term dependency within the time series which makes it hard to improve the performance of the model. To solve the problem, Long Short-Term Memory (LSTM) network has been widely used in HAR because of its advantages in extracting long-term dependence within time series [20,21]. Mohib et al. [22] proposed a stacked LSTM network for recognizing human behaviors using smartphone data. The accuracy of the proposed network is 93.13%. Zhao et al. [23] proposed a residual BiLSTM to address the HAR problem. The residual connection built between the stacked cells can avoid the gradient vanishing problem. Alawneh et al. [24] compared the results of the LSTM and BiLSTM models on the sensor-based HAR dataset. The results showed that the BiLSTM outperforms the LSTM in the recognition accuracy.

A lot of recent work on HAR focused on the hybrid model of CNN and RNN. Nafea et al. [25] proposed a model using CNN with varying kernel dimensions along with BiLSTM to obtain features at different resolutions. It has a high accuracy on the WISDM and UCI datasets. Nan et al. [26] proposed a multichannel CNN-LSTM network for smartphone-based HAR in elderly people. Fifty-three elderly people participated in the experiment, and the results showed that the proposed network performed better than CNN and CNN-LSTM. In [27], four deep learning hybrid models composed of CNN and RNN were studied to recognize complex activities. Experimental results show that CNN-BiGRU performs better than several other models.

In addition, some papers introduced the self-attention to HAR. Abdel et al. [28] proposed a dual-channel network composed of convolutional residual network, LSTM, and attention mechanism. The accuracy of the proposed network on WISDM reached 98.9%. Mahmud et al. [29] employed self-attention to identify human activities, and the F1 score of the model is 96%. Although many DL studies have achieved great success in the field of HAR, their performance is not the best due to neglecting to exploit both spatial and temporal information of sensor data. Some networks [6,25,29] are relatively complex, making them difficult to run in the devices which have limited computer sources and memory spaces. To solve these problems, in this study, we proposed a new DL model which cascades a residual network with BiLSTM. Firstly, we use a residually connected convolutions (ResNet) [30] to extract the spatial features of sensor data. Then we use BiLSTM to obtain forward and backward dependencies of feature sequence.

The primary contributions of this work are as follows:

(1) A new model, combining the ResNet with BiLSTM, is proposed to capture the spatial and temporal feature of sensor data. The rationality of this model is explained from the perspective of human lower limb movement and the corresponding IMU signal.
(2) We introduce the BiLSTM into ResNet to extract the forward and backward dependencies of feature sequence which is useful to improve the performance of the network. We analyze the impact of model parameters on classification accuracy. The optimal network parameters are selected through experiments.
(3) An HAR dataset, in which the human activity data are collected by a self-developed IMU board, was made. The IMU board is attached to human shank to collect the activity data of the human lower limbs. Our model performs well on this dataset. The proposed model was also tested on both the WISDM and PAMA2 HAR datasets and outperforms existing solutions.

The rest of this paper is organized as follows. In Section 2, the proposed mode is described. Section 3 describes the collection of sensor data, the public HAR dataset, experimental setup, experimental results and discussion. Section 4 is the conclusion of this paper.

## 2. Proposed Approach

**Figure 1:**
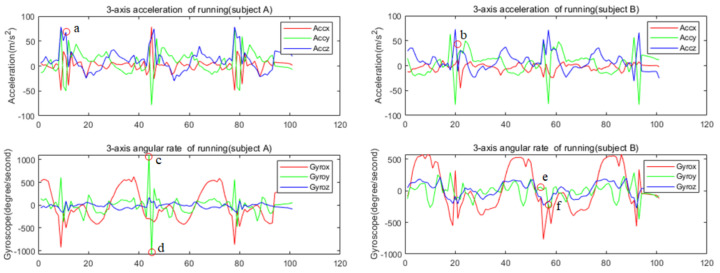
IMU signal of different people on running. a, b, c, d, e, f: the different moment of IMU signal when two people are running.

With the massive application of MEMS IMU in the smartphone and wearable systems, the HAR is gradually shifting from image-based to sensor-based. Figure 1 shows the signal of IMU attached to the shanks on different people while running. The traditional HAR method firstly calculates the features of IMU signal in a period, such as the mean, variance, and maximum value of sensor data in the sliding window, or the correlation coefficient of different channel signals. Then, the calculated features are used to judge the activity category by some preset thresholds, or the calculated features are put into a machine learning model for training and classification. As we know, for the same activity, there are differences in different people’s movements and differences in one person’s movement at different times; therefore, the calculated features are quite different. The calculated features of different actions by traditional methods tend to overlap. As shown in Figure 1, the signal amplitude at a certain point (c, d, e, f) in the running cycle of two people is different, obviously, which leads to large differences in the hand-crafted features. Therefore, it is hard to recognize the human activity using such features and more powerful feature extraction methods are needed.

ResNet [30] is an important improved CNN model with powerful local spatial feature extraction capability widely used in the field of image recognition. It can also be used to extract the local features between different channels of IMU signal in a small sampling segment, that is, the local spatial features of IMU signal. However, the human motion, especially the motion of the lower limbs, is a non-rigid motion. There are some irregular changes in the IMU signal due to the movement of the lower limbs of the human body within a short period. For example, there is an irregular spike at point a for subject A, but it is smooth at point b for subject B in Figure 1. Only extracting the spatial features of the IMU signal may easily lead to false recognition. For a long time, the sensor signal is relatively flat and periodic due to the stability and periodicity of human gait. We can obtain the dependence of the sensor signal over a long time to improve the recognition accuracy. Therefore, we consider using LSTM to extract the long-term dependency of IMU signal.

**Figure 2:**
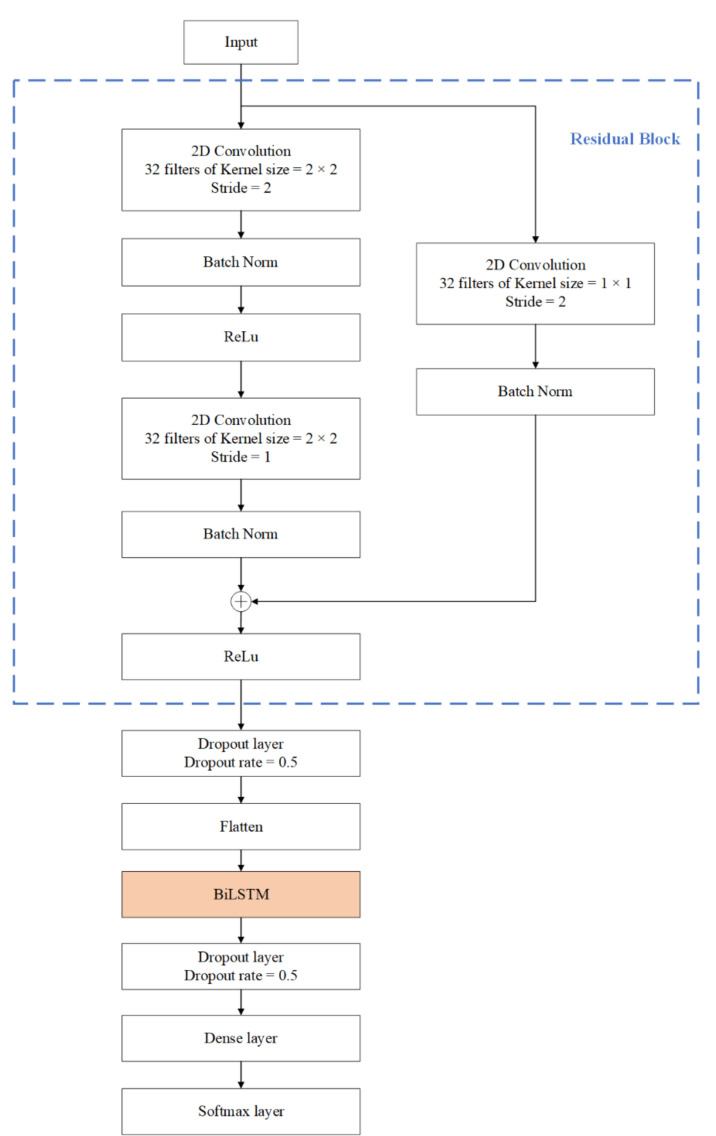
Frame diagram of the proposed model.

BiLSTM is a special LSTM that can extract both forward and backward dependence on the time sequences [23]. We proposed a new model by merging the ResNet and BiLSTM based on the above analysis of the characteristics of human limb movement and IMU signal. The architecture of our proposed model is presented in Figure 2. As shown in the figure, the input data are firstly processed by the residual block to extract the local spatial features of the data. Then, the flattened features are fed into BiLSTM. There is a dropout layer followed by the BiLSTM to avoid overfitting. After a dense layer, a Softmax layer is used for yielding a probability distribution over classes at the end of the model.

### 2.1. Spatial Feature Extraction Based on ResNet

Due to the differences of the movements of the lower limbs when different individuals exercise and the complexity of human movements, the extracted hand-crafted features of different human activities are easy to overlap. It is difficult to separate these features by threshold or machine learning model. Manual features that are easy to distinguish are also difficult to design. Traditional methods use the manual features to recognize human activities, and the effect is not good. The CNN model can automatically extract the local spatial features of the sensor signal by learning lots of samples. The powerful capability of feature extraction improves the accuracy of HAR. It has been widely used in sensor-based HAR. However, increasing the number of convolutional layers in the model results in accuracy saturation. The ResNet is proposed to solve this problem. In the shallow network, the residual module can be also beneficial to improve network performance [30]. As shown in Figure 2, the residual module is composed of two convolutional layers connected in sequence, and a parallel skip link is added. In order to obtain the spatial features of the different channels of sensor signal, the two-dimensional convolutional residual network is used. The first convolution layer is designed with 32 kernels of size 2 × 2. The stride length of the convolution window is 2. A Batch Norm (BN) layer is added to this convolutional layer, to speed up the training process, and to avoid problems of covariate shift. The BN layers are followed by the ReLu activation function, which has the advantage of avoiding gradient disappearance. The second convolution layer has the same parameters except the stride length is 1. In order to make the dimension of output of the two convolutional layers consistent with the original input dimension, the same two-dimensional convolution is performed in the parallel skip linking. Let the input of residual block be x, the output can be expressed as

(1) y=f(x)+h(x)

where f(x) represents the mapping learned by the stacked convolutional layers and h(x) represents the mapping learned by the shortcut connection part. When the shortcut connection part is added, the residual network is prone to learn constant mappings after the network reaches its optimal performance, thus at least not deteriorating the network performance, while more parameters allow for a greater model fitting potential. The model parameters are described later.

### 2.2. BiLSTM Layer

**Figure 3:**
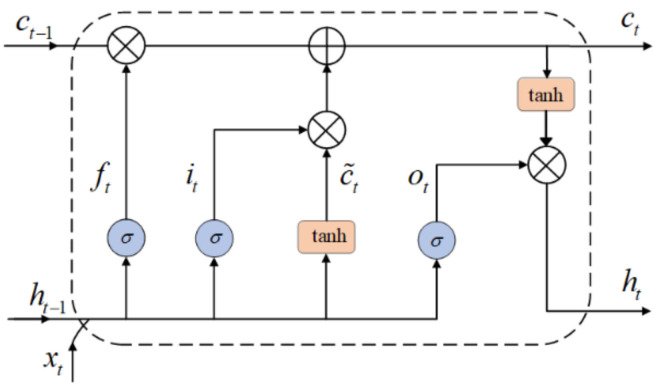
Long short-term memory cell [32].

As mentioned above, only extracting the local spatial features of the sensor signal of human activity is not enough for HAR. The RNN model has the ability to capture temporal information from time sequences. However, Bengio et al. [31] inform that RNN networks can recognize the data for only a moment, owing to the vanishing and exploding gradient issue. LSTM is a special type of RNN that solves the problem of long-time dependence of time series due to its special memory cells [32]. In this study, we use a special LSTM named BiLSTM to analyze the local spatial feature sequence to obtain the long-term regularity of the sensor signal. Figure 3 shows the cells of LSTM.

LSTM is implemented through three gates: input gate, forget gate and output gate. An LSTM unit can be defined and explained as follows, where U, W is the weight matrix, and b is the bias term:

(2) $$i_{t}=\sigma (U_{i}\cdot x_{t}+W_{i}\cdot h_{t-1}+b_{i})$$

where, $i_{t}$ is the input gate at time t, ⋅ is the matrix multiplication, σ is the sigmoid function, $x_{t}$ is the input data at time t, and $h_{t-1}$ is the output of the previous LSTM unit. The input gate determines which information in the previous unit needs to be updated.

(3) $$f_{t}=\sigma (U_{f}\cdot x_{t}+W_{f}\cdot h_{t-1}+b_{f})$$

where, $f_{t}$ represents the forget gate which calculates the importance of the information and forgets some old information.

(4) $${\tilde{c}}_{t}=\tan h(U_{c}\cdot x_{t}+W_{c}\cdot h_{t-1}+b_{c})$$

(5) $$c_{t}=f_{t}⊙c_{t-1}+i_{t}⊙{\tilde{c}}_{t}$$

The candidate state ${\tilde{c}}_{t}$ is calculated with the tanh function as depicted in Equation (3). Then, the present cell state is computed as expressed in Equation (4), where, ⊙ denotes element multiplication.

(6) $$o_{t}=\sigma (U_{o}x_{t}+W_{o}h_{t-1}+b_{o})$$

(7) $$h_{t}=o_{t}⊙\tan h(c_{t})$$

In Equation (6), the output gate $o_{t}$ is calculated. In Equation (7), $h_{t}$ is the output of LSTM unit.

**Figure 4:**
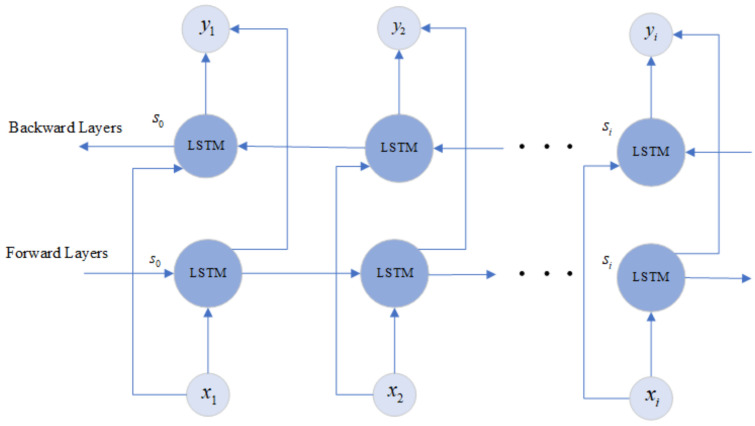
Frame diagram of the BiLSTM.

Baseline LSTM predicts the human current activity based only on former data. It is obvious that some information may be lost if the data are considered on only one direction. The BiLSTM is made up of two LSTM layers in two directions. As shown in Figure 4, the output of the BiLSTM is determined by the LSTM in the forward layers and backward layers together. In the BiLSTM, the output layer $y_{t}$ is expressed as follow [33]:

(8) $$y_{t}=\left[{\vec{h}}_{t},{\overset{\leftarrow }{h}}_{t}\right]$$

where, the ${\vec{h}}_{t}$ and ${\overset{\leftarrow }{h}}_{t}$ represent the forward and backward results of LSTM units. The output $y_{t}$ is formed by concatenating these two LSTM units.

## 3. Experiments Results and Discussion

### 3.1. Data Collection

#### 3.1.1. The Collection of Homemade Dataset

**Figure 5:**
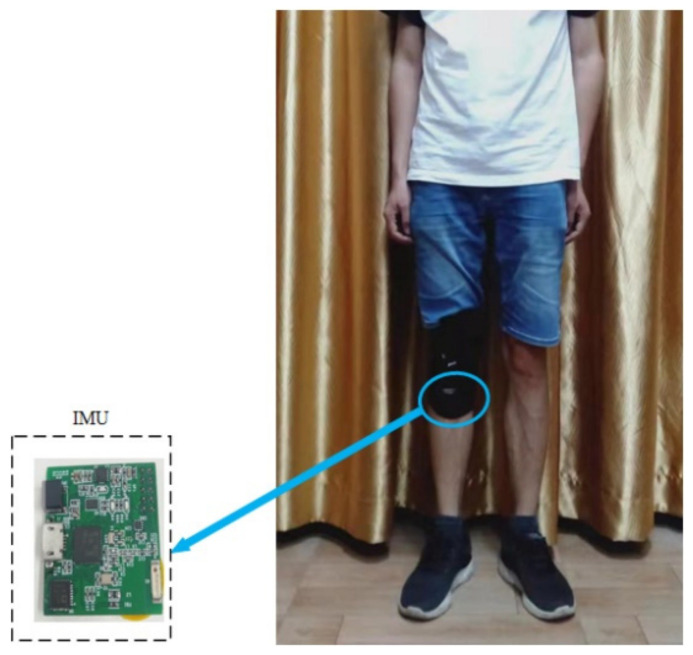
Fixed position of IMU module.

Unlike most studies in which the devices such as smartphones are fixed to a person’s waist to collect human activity data, the self-developed IMU module is fixed to the human body below the knee at the shin in this paper, as shown in Figure 5. Regarding human activities, lower limb activities have attracted much attention. For example, runners want to know their amount of exercise. The monitoring of walking time of patients with lower extremity diseases during rehabilitation is also considered. Therefore, we mainly consider the recognition of human lower limb activities. The sensor is placed under the human knee to directly obtain the movement information of the human body’s lower limbs. The sensor module can be fixed together with commonly used knee pads and is very convenient to use in the real applications.

This IMU module is powered by a lithium battery (3.7 V, 230 mah), which can be charged by USB. A 9-axis inertial sensor (MPU9250) is integrated on the module, including a 3-axis accelerometer, a 3-axis gyroscope, and a 3-axis magnetometer. There is a processor (Texas Instruments, CC2642) on the module, which integrates the ARM Cortex-M4F microcontroller unit. The size of our module is about 3.5 cm (length) by 2.5 cm (width) by 1 cm (height). As the magnetometer in the IMU module may be affected by the surrounding environment, the accelerometer and gyroscope are used here. The 3-axis acceleration and three-axis angular rate data were collected by the accelerometer and gyroscope at a sampling frequency of 50 Hz. The dynamic range of the accelerometer and gyroscope output data were set to ±8 g and ±2000 dps, respectively. The sensor data were firstly stored in the Flash memory of the IMU module, and then the data were transmitted to the computer through serial port for processing.

**Figure 6:**
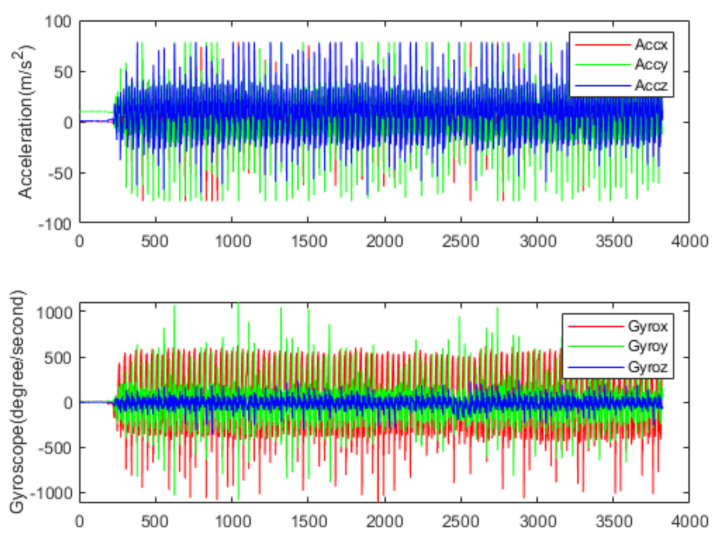
Data collected by accelerometer and gyroscope on running.

**Figure 7:**
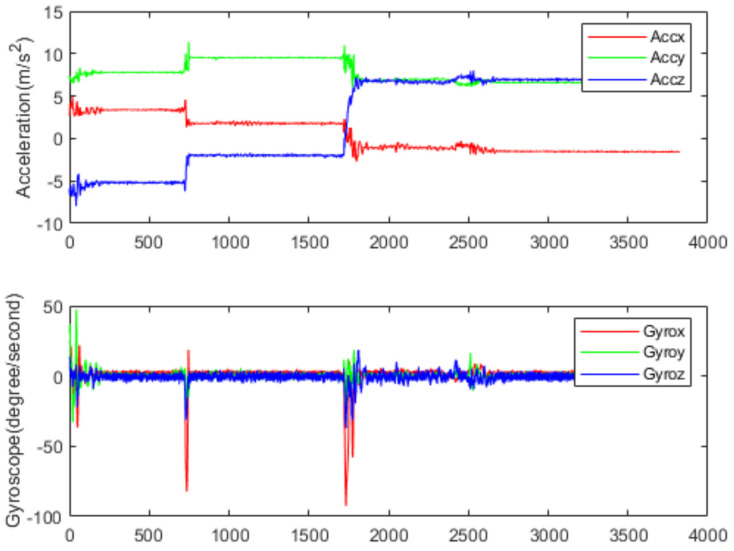
Data collected by accelerometer and gyroscope on sitting.

Figure 6 and Figure 7 show the acceleration and angular rate data collected under the running and sitting within 77 s, respectively. From Figure 6, we can see that the human activities of the running are intense, and the fluctuation range of both acceleration and angular rate is relatively large. In this paper, five volunteers (age range from 22 to 32) from the laboratory participated in the data collection. Each volunteer was asked to do six classes of human activities: sitting, standing, walking, running, going upstairs and going downstairs. Finally, a dataset containing 130,056 samples was made. The detailed information is shown in Table 1. The whole dataset was divided into training and validation sets, with 70% for training and 30% for validation.

#### 3.1.2. The Public Dataset

The WISDM dataset includes 1,098,209 samples [7]. Thirty-six subjects used a smartphone placed in their trouser pockets to complete six daily activities. The data were collected by an accelerometer in the mobile phone at a sampling frequency of 20 Hz. Walking (38.6%), jogging (31.2%), upstairs (11.2%), downstairs (9.1%), sitting (5.5%), and standing (4.4%), were the collected activities.

The PAMAP2 dataset contains 18 daily activities, including 12 protocol activities (walking, running, rope skipping, vacuum cleaner cleaning, etc.) and six optional activities (watching TV, folding clothes, etc.). The activity information of nine subjects was collected by heart rate meter, three IMU modules and thermometer. The three IMU modules were placed on different positions on the subjects: one IMU on the arm, one IMU on the chest, one IMU on the ankle. The sampling frequency of the sensor is 100 Hz [34].

### 3.2. Data Preprocessing

**Figure 8:**
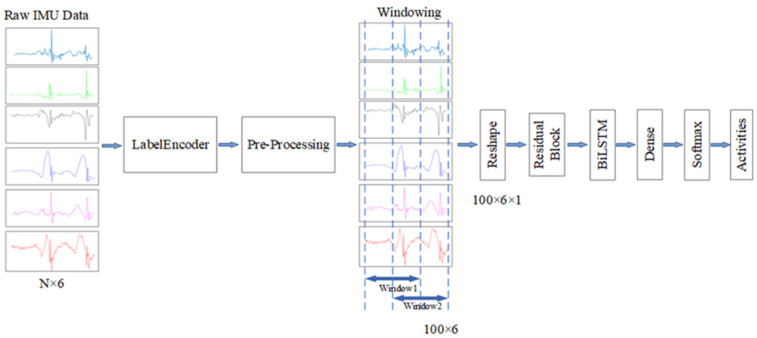
Frame diagram of the sensor raw data processing flow.

Figure 8 is the sensor raw data processing flow. Accelerometers and gyroscopes use the same frequency 50 Hz sampling to obtain time series. After a period of sampling, the sequence obtained by the six-axis IMU can be represented as

(9) $$I=\{X_{i},i=1,2,\ldots ,N\},\;X_{i}=(a_{x,i},\;a_{y,i},\;a_{z,i},\;g_{x,i},\;g_{y,i},\;g_{z,i})$$

where $\left(a_{x,i},a_{y,i},a_{z,i}\right)$ is the triaxial data sampled by the accelerometer and $\left(g_{x,i},g_{y,i},g_{z,i}\right)$ is the triaxial data sampled by the gyroscope. A label $y_{i}$ is assigned to each vector $X_{i}$ to obtain $I^{\prime }=\{(X_{i},y_{i}),i=1,2,\ldots ,N\}$.

As the scale of raw data from different sensors varies greatly, the unprocessed data with big fluctuation affected the performance of network [35]. In addition, the standardization of the data range is helpful to find the global optimal solution in training compared the raw data. Therefore, it is necessary to standardize the data. In this paper, the mean and standard deviation are used to standardize the acceleration data and gyroscope data. The x axis data of accelerometer are standardized according to Equation (10).

(10) $$a_{x}^{\prime }=\frac{a_{x}-\mu_{x}}{\sigma_{x}}$$

where, $a_{x}$ is the x axis data of accelerometer, and $\mu_{x}$, $\sigma_{x}$ represents the average value and standard deviation of all the x axis raw data of accelerometer, respectively. The output data of the other five axes are standardized in a similar way.

In the current research, the segmentation of basic actions mostly adopts a fixed window size of the sliding window method with fixed overlap coverage rate. The cycle of dynamic actions such as walking is 1–2 s. In this paper, we also use a sliding window with a window length of 100 and an overlap coverage of 50% to segment human actions.

### 3.3. Experimental Environment

The network in this paper was trained on a computer equipped with Intel Core i9-9900 CPU, 16 GB RAM and a graphics processor (GPU) (NVIDIA GeForce GTX 1060 with 6 GB memory). The algorithm was implemented using python 3.8 based on Google’s open source deep learning framework Tensorflow 2.3.0, and the development environment of experiments is Pycharm on a 64-b version of Windows 10. The GPU is used to speed up the training and testing of the model.

### 3.4. Evaluation Index

To evaluate the performance of the proposed model for HAR, the followed metrics [36] were used for evaluation generally.

(11) $$\mathrm{Accuracy}=\frac{TP+TN}{TP+FN+FP+TN}$$

(12) $$\mathrm{Precision}=\frac{TP}{TP+FP}$$

(13) $$\mathrm{Recall}=\frac{TP}{TP+FN}$$

(14) $$F_{1}-\mathrm{score}=\frac{2\times Precision\times Recall}{Precsion+Recall}$$

(15) $$\mathrm{Fw}=\sum_{i}2\times \omega_{i}\frac{Precision_{i}\times Recall_{i}}{Precision_{i}+Recall_{i}}$$

where TP, TN are the number of true and false positives, respectively, and FN, FP corresponds to the number of false negatives, false positives. $\omega_{i}$ is the proportion of samples of class i.

### 3.5. The Optimal of Hyperparameters

#### 3.5.1. The Optimal of Model Parameters

In order to obtain the optimal parameters of the proposed model, i.e., the size of convolution kernels, the number of convolution kernels, the number of LSTM units, and the dropout ratio, we adjust them in turn and finally select the appropriate parameters.

**Figure 9:**
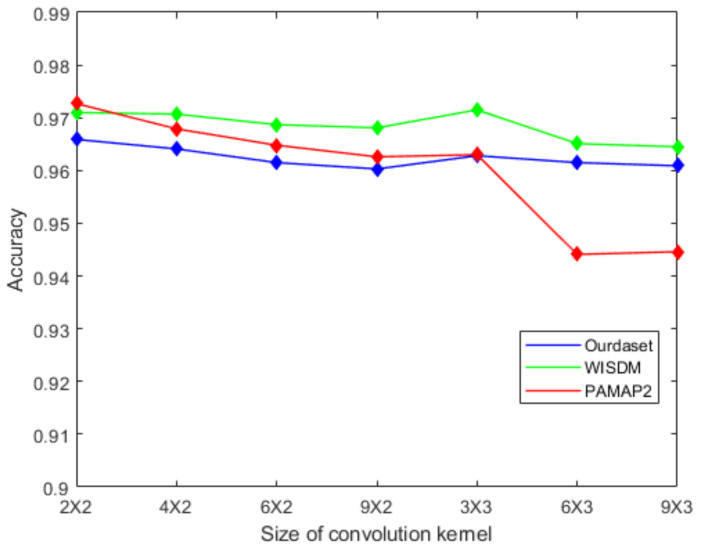
The effect of convolution kernel size on recognition accuracy.

Firstly, we analyze the effect of the size of the convolution kernel on the classification accuracy. Seven different convolution kernel sizes are set to test. Figure 9 shows the effect of convolution kernel size on recognition accuracy. It can be seen that the network accuracy is degraded when the size of the convolution kernel increases. We choose the size of the convolution kernel to be 2 × 2 because this size has the best recognition accuracy.

**Figure 10:**
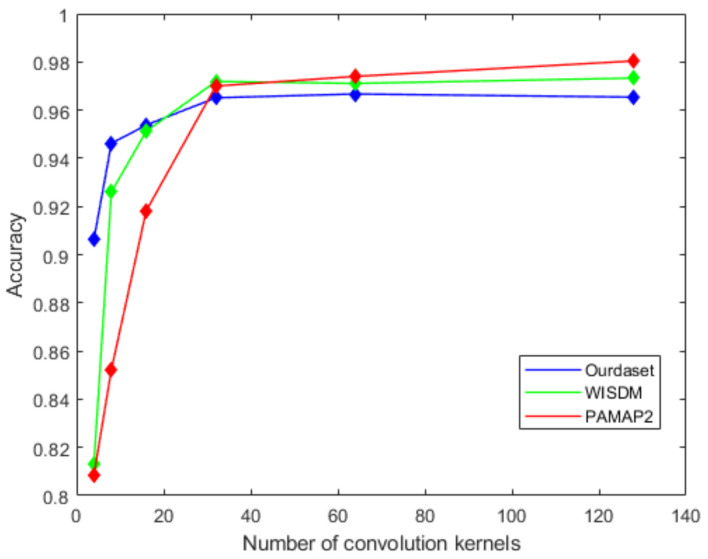
The effect of the number of convolution kernels on recognition accuracy.

On the basis of the optimal size of convolution kernel, we analyze the effect of the number of convolution kernels on the recognition accuracy. We set the number of convolution kernels to 4, 8, 16, 32, 64, and 128 and record the accuracy. It can be seen from Figure 10 that as the number of convolution kernels increases, the recognition accuracy is improved. When this parameter increases to 32, the improvement of accuracy is very small. The accuracy corresponding to the values of 64 and 32 is basically the same. As we know, a larger number of convolution kernels will increase the model size and require higher training costs. Finally, we choose 32 as the number of convolution kernels.

**Figure 11:**
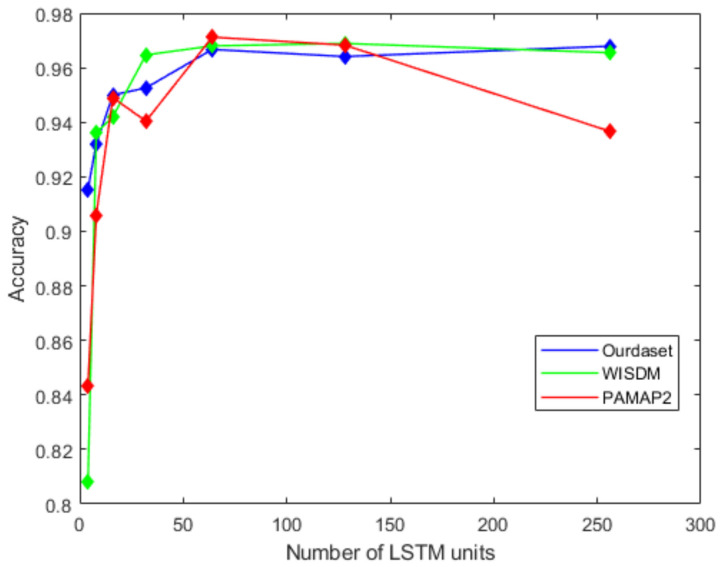
The effect of the number of LSTM units on recognition accuracy.

**Figure 12:**
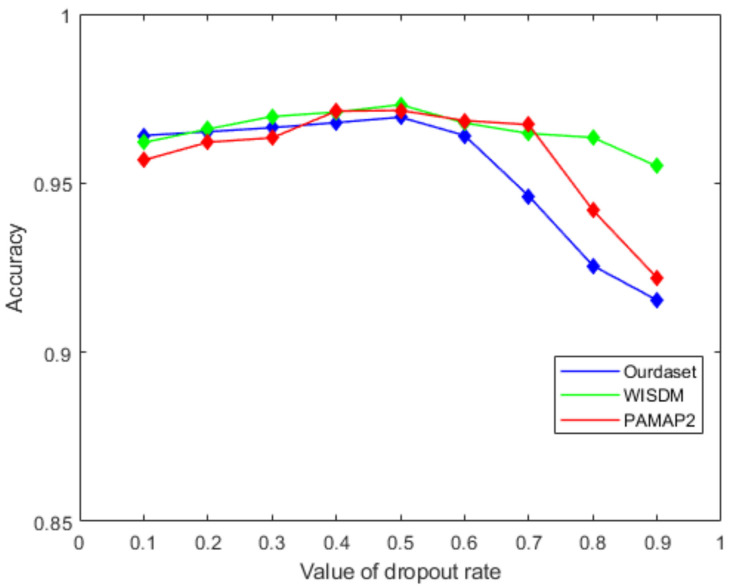
The effect of the value of dropout rate on recognition accuracy.

Figure 11 shows the effect of the number of LSTM units in the BiLSTM module on the recognition accuracy. It can be seen from the figure that as the number of LSTM units increases in the beginning, the recognition accuracy will also improve. When the number of LSTM units increases to 128, the accuracy of the proposed network gets worse. In this paper, we choose 64 as the number of model LSTM units. Similarly, we select the dropout ratio from 0.1 to 0.9 in step of 0.1, and test the accuracy of the network in turn. Figure 12 shows that the best network accuracy is obtained when the dropout ratio is 0.5.

#### 3.5.2. Hyperparameters of the Model Trained

The proposed model was trained by minimizing the cross entropy using the Adam optimizer as in [37]. As in [38], the learning rate is firstly set to several constants, i.e., 0.00001, 0.0001, 0.001, 0.01. Then, we find that the possible optimal learning rate may be between 0.0001 and 0.001 for our dataset and the WISDM dataset by comparing the accuracies. For the PAMAP2 dataset, the possible optimal learning rate will be between 0.00001 and 0.0001. We look for the optimal parameter between [0.0001, 0.001] with a step of 0.0001 for our dataset and WISDM dataset. Similarly, we seek the optimal parameters on [0.00001, 0.0001] with a step of 0.00001 for the PAMAP2 dataset. Finally, we select the 0.0003, 0.0006, 0.00003 as the learning rate for our dataset, WISDM, and PAMAP2, respectively. The batch size used to train the model on three datasets is 64. The number of repeated trainings of the model is 80. The hyperparameters used for model training are shown in Table 2.

### 3.6. Experiment Result

**Figure 13:**
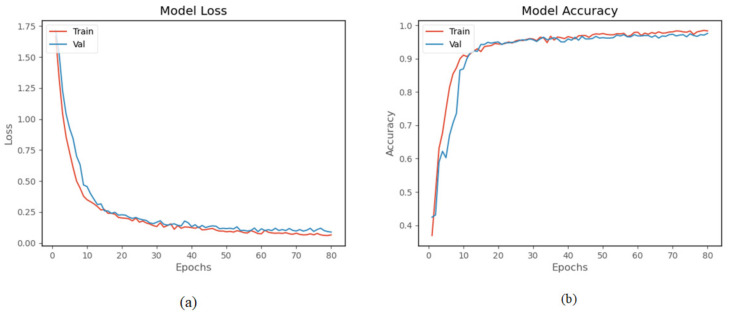
Experiment results on the homemade lower extremity activity dataset: (**a**) the training loss and validation loss, (**b**) the training accuracy and validation accuracy.

The proposed model was trained and tested using a homemade human activity dataset. The model was trained 80 times. The experimental results in Figure 13a show that both training loss and validation loss decrease as the number of training times increases. The two curves are very close after 20 times, and after 70 times, the amplitude of the curves basically remain stable. Figure 13b shows the accuracy curve of the model on the homemade dataset, and the accuracy of the validation set finally reached 96.95%. The result indicates that the model has good performance on the homemade human activity dataset.

Table 3 shows the confusion matrix for the proposed model on our dataset. In the table, HA1 = running, HA2 = walking, HA3 = standing, HA4 = sitting, HA5 = going upstairs, HA6 = going downstairs, PRC: precision, RCL: recall, and F1S: $F_{1}-score$. The diagonal element of the confusion matrix represents the number of correctly recognized activity, and the off diagonal element represents the number of incorrectly recognized activities. There is some confusion between the going upstairs and going downstairs. This is mainly due to the great similarity between the two human activities, resulting in the similar data collected by accelerometer and gyroscope. HA1–HA4 is confused. It can be seen from Figure 5 that the pedestrian is in a static state at the beginning of running. The signal at this stage is similar to the signal of sitting. In addition, the amount of running data in the dataset are relatively small, which makes the model less sensitive to this mode. Therefore, the trained model incorrectly identifies a few running samples as sitting. The table lists the indicators for each category. Among them, the $F_{1}-score$ of walking and sitting is up to 0.99. The lowest $F_{1}-score$ is running, only above 0.92. The $F_{w}$ is 0.9712, indicating that the network has good classification performance.

**Figure 14:**
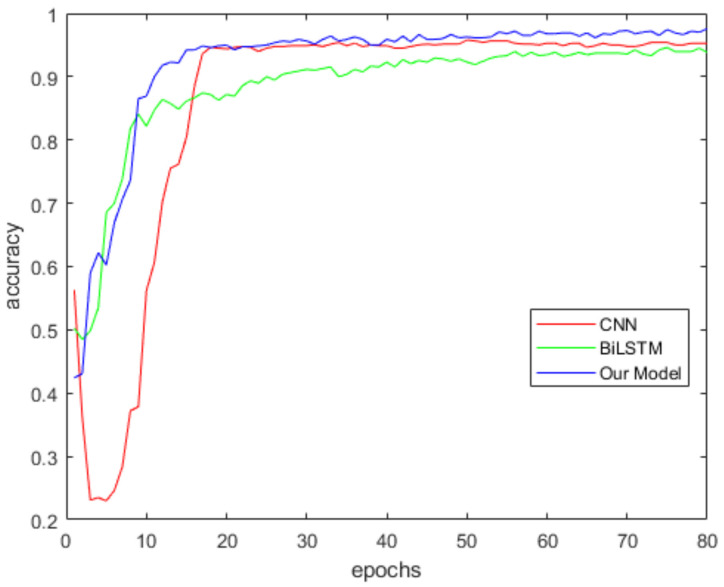
Comparison between the proposed model and two baseline models on the homemade dataset.

We compared the accuracy of the proposed model and two baseline models on the homemade dataset. The test was performed 10 times. Each time the dataset was randomly divided into the training set (70%) and the validation set (30%). CNN, BiLSTM and our model were tested in turn for each test. Then, we saved the accuracy of each model on the validation set and averaged it. In the experiment, the CNN has two convolution layers. Each layer has 32 convolution kernels with a size of 2 × 2. The number of hidden units of BiLSTM model is 64. The hyperparameters of training are shown in Table 1. Figure 14 shows the average accuracy of our model, CNN and BiLSTM on the homemade dataset. As shown in the figure, the accuracy of the proposed model is higher than CNN and BiLSTM. The proposed model can extract comprehensive feature information from human activity data, so the accuracy is better than the CNN model and BiLSTM model.

### 3.7. Model Performance on Public Datasets

Although the proposed model has achieved good results on our homemade dataset, to comprehensively verify the performance of the model, the public WISDM and PAMAP2 datasets were tested in our experiment.

#### 3.7.1. Performance on WISDM Dataset

**Figure 15:**
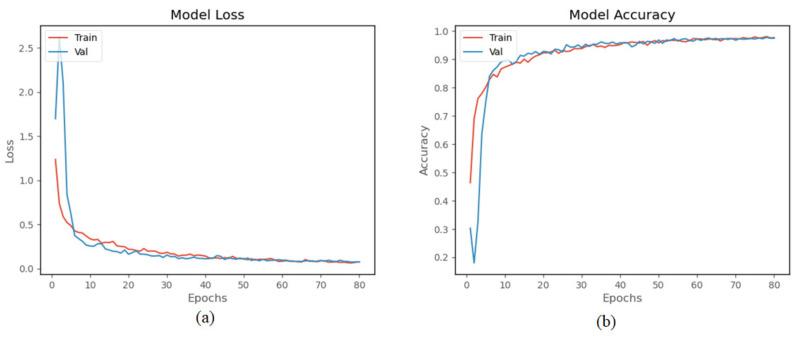
Results of the model on WISDM dataset: (**a**) training loss and validation loss, and (**b**) training accuracy and validation accuracy.

**Figure 16:**
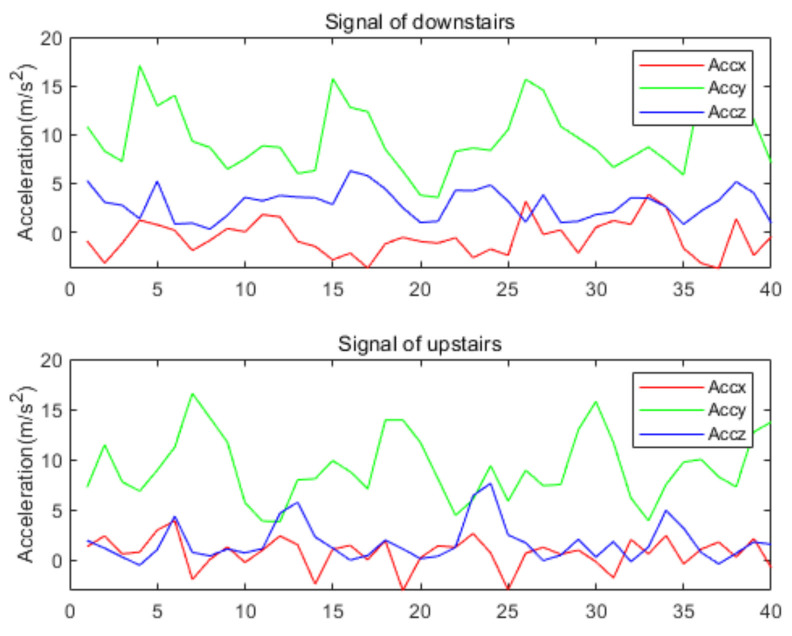
Comparison of the signal of upstairs and downstairs.

The WISDM dataset was collected by a single accelerometer in the smartphone placed in the trouser pocket with a sampling frequency of 20 Hz [7]. Like the preprocessing of our homemade dataset, the sliding window size is set to 40, and the overlapping area accounts for 50%. The whole dataset is divided into training set (70%) and validation set (30%). The hyperparameters of training are set as Table 2. As shown in Figure 15, the proposed model of accuracy is 97.32% on WISDM. Table 4 is the confusion matrix on the WISDM dataset, where HA1 = downstairs, HA2 = jogging, HA3 = sitting, HA4 = standing, HA5 = upstairs, and HA6 = walking. The table shows that the highest $F_{1}-score$ of HA2, HA3, HA4, and HA6 reached 0.99, while HA1–HA5 is easily confused as the acceleration producing by these activities is similar. Figure 16 shows the signal of upstairs and downstairs from WISDM. As shown in the figure, there is no obvious difference between the signals for downstairs and upstairs. The amplitude and frequency of the signals of the two activities are similar. The $F_{w}$ is 97.31%.

#### 3.7.2. Performance on PAMAP2 Dataset

**Figure 17:**
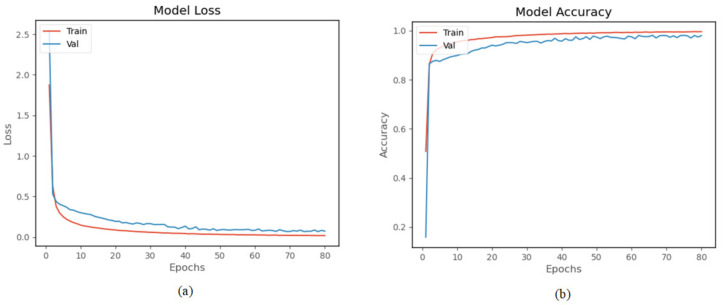
Results of the model on PAMAP2 dataset: (**a**) training loss and validation loss, and (**b**) training accuracy and validation accuracy.

In this paper, the data of 18 channels collected by three IMU modules in the PAMAP2 dataset are selected to test the proposed model. The data from subject 5 and 6 are used as the validation set, and the data from the other subjects are used as the training set. The sliding window size is set to 100 and the overlapping area accounts for 50%. The hyperparameters of training are shown in Table 2. Twelve protocol activities in the dataset were used to test. As shown in Figure 17, the accuracy of the model in this dataset is 97.15% and $F_{w}$ is 97.35%. Table 5 shows the confusion matrix of the proposed model on the PAMAP2 dataset. In the table, HA1 = lying, HA2 = sitting, HA3 = standing, HA4 = walking, HA5 = running, HA6 = cycling, HA7 = Nordic walking, HA8 = ascending stars, HA9 = descending stars, HA10 = vacuum cleaning, HA11 = ironing, and HA12 = rope jumping.

### 3.8. Comparison with Existing Work

In the following, we compare the proposed model in this paper with the related work in recent years. The selected works are based on the WISDM and PAMAP2 datasets in Table 6. It could be noted that the recognition accuracy and $F_{w}$ of our model have improved compared with the relevant work. Firstly, our model is more accurate than CNN, TSE-CNN and SC-CNN, because these models ignored the long-time dependence of sensor feature data. Secondly, our model is more accurate than the hybrid model of CNN and LSTM, or hybrid model of CNN and GRU, because BiLSTM can extract the forward and backward information between time series, while LSTM or GRU only considers the forward information. In addition, the accuracy and $F_{w}$ of our model are higher than the model based on attention mechanism. It reflects that the performance of our model is better than most existing models. In terms of the number of model parameters, our model is also smaller than those in [6,29,39]. Smaller models are easier to apply in mobile devices.

## 4. Conclusions

A model incorporating residual network and BiLSTM is proposed for the problem of HAR. The model can comprehensively extract the local spatial features of the sensor data and bi-directional long-term dependence within the spatial features. A new homemade HAR dataset in which the human lower limb activity data are collected by a self-developed IMU module was constructed. The proposed model was tested using the homemade HAR dataset with the accuracy of 96.95%. We tested the proposed model with WISDM and PAMAP2 datasets, and the results show that our model accuracy is 97.32% and 97.15%, respectively. Through the comparison with the previous work, we find that the proposed model obviously achieves improved accuracy and $F_{w}$. In addition, our model has fewer parameters than some existing models. We believe that our work has important application prospects in the fields of physical training, health management, human lower limb rehabilitation therapy, etc. The model will be further improved in the future to enhance the accuracy of the model.

## Figures and Tables

**Table 1:** Activities of our dataset.

Activity	Sitting	Standing	Walking	Running	Going Upstairs	Going Downstairs
	42.4%	12.7%	14.4%	9.6%	10.9%	10%

**Table 2:** Hyperparameters of the model trained.

Hyperparameters	Value
Loss function	Cross entropy
Optimizer	Adam
Batch size	64
Learning rate	0.0003 (for our dataset)
	0.0006 (for WISDM)
	0.00003 (for PAMAP2)
Training times	80

**Table 3:** Confusion matrix for the proposed model on our dataset.

Activity	HA1	HA2	HA3	HA4	HA5	HA6	RCL	F1S
HA1	65	1	2	5	1	1	0.87	0.92
HA2	0	112	0	0	0	0	1.00	0.99
HA3	0	1	97	1	0	0	0.98	0.95
HA4	0	0	3	328	0	0	0.99	0.99
HA5	1	0	3	0	77	4	0.91	0.94
HA6	1	0	1	0	1	75	0.96	0.95
PRC	0.97	0.98	0.92	0.98	0.97	0.94		

**Table 4:** Confusion matrix of model on WISDM dataset.

Activity	HA1	HA2	HA3	HA4	HA5	HA6	RCL	F1S
HA1	455	3	0	1	29	11	0.91	0.90
HA2	10	1919	0	0	5	7	0.98	0.99
HA3	0	0	68	0	1	0	0.98	0.99
HA4	0	0	0	53	0	0	1.00	0.99
HA5	36	12	0	0	469	10	0.88	0.90
HA6	5	0	0	0	7	2049	0.99	0.99
PRC	0.89	0.99	1.00	0.98	0.91	0.98		

**Table 5:** Confusion matrix of model on PAMAP2 dataset.

	HA1	HA2	HA3	HA4	HA5	HA6	HA7	HA8	HA9	HA10	HA11	HA12	RCL	F1S
HA1	493	0	0	0	0	0	0	0	0	0	0	0	1.00	0.96
HA2	46	504	10	0	0	0	0	0	0	0	0	0	0.90	0.95
HA3	0	0	459	2	0	0	0	0	0	0	0	0	1.00	0.99
HA4	0	0	0	667	0	0	0	0	0	0	0	0	1.00	0.99
HA5	0	0	0	6	483	24	0	0	0	0	0	0	0.94	0.97
HA6	0	1	0	1	3	490	17	0	0	0	0	0	0.96	0.96
HA7	0	0	0	0	0	0	547	0	0	0	0	0	1.00	0.98
HA8	0	0	0	0	0	0	0	295	2	0	0	0	0.99	0.98
HA9	0	0	0	0	0	0	0	0	246	11	0	0	0.93	0.96
HA10	0	0	0	0	0	0	0	0	0	500	9	0	0.98	0.96
HA11	0	0	0	0	0	0	0	0	0	19	669	0	0.97	0.98
HA12	0	0	0	0	0	0	0	0	0	0	0	160	1.00	1.00
PCR	0.91	1.00	0.98	0.99	0.99	0.95	0.97	0.97	0.99	0.94	0.99	1.00		

**Table 6:** Comparison with existing work.

Dataset	Reference	Accuracy	*F_w_*	Params
WISDM	CNN [15]	93.32%	-	-
	TSE-CNN [17]	95.7%	94.01%	9223
	SC-CNN [6]	97.08%	-	1,176,972
	CNN-GRU [21]	97.21%	97.22%	-
	LSTM-CNN [40]	95.01%	95.85%	62,598
	Our Model	97.32%	97.31%	71,462
PAMAP2	CNN-GRU [21]	95.27%	95.24%	-
	CNN [41]	91%	91.16%	-
	Self-Attention [29]	-	96%	428,072
	CNN-Attention [39]	93.16%	-	3,510,000
	Our Model	97.15%	97.35%	185,376
